# Tracking Short-Range Attraction and Oviposition of European Grapevine Moths Affected by Volatile Organic Compounds in a Four-Chamber Olfactometer

**DOI:** 10.3390/insects11010045

**Published:** 2020-01-08

**Authors:** Anna Markheiser, Margit Rid, Sandra Biancu, Jürgen Gross, Christoph Hoffmann

**Affiliations:** 1 Institute for Plant Protection in Fruit Crops and Viticulture—Zoology and Integrated Plant Protection, Julius Kühn-Institut (JKI), Federal Research Centre for Cultivated Plants, Geilweilerhof, 76833 Siebeldingen, Germany; sandra.biancu@julius-kuehn.de (S.B.); christoph.hoffmann@julius-kuehn.de (C.H.); 2 Institute for Plant Protection in Fruit Crops and Viticulture—Applied Chemical Ecology, Julius Kühn-Institut (JKI), Federal Research Centre for Cultivated Plants, Schwabenheimer Str. 101, 69221 Dossenheim, Germany; margit.rid@julius-kuehn.de (M.R.); juergen.gross@julius-kuehn.de (J.G.); 3 Plant Chemical Ecology, Technical University of Darmstadt, Schnittspahnstr. 4, 64287 Darmstadt, Germany

**Keywords:** *L. botrana*, *E. ambiguella*, grape berry moth, VOC, host plant choice, behavior, egg deposition, EAG, kairomones, pheromones

## Abstract

The identification of volatile organic compounds (VOCs) leading to short-range attraction and oviposition of the European grapevine moth *Lobesia botrana* and European grape berry moth *Eupoecilia ambiguella* (Lepidoptera: Tortricidae) is crucial in order to establish bait-based decision support systems for control of these pests. Therefore, we developed a method to measure the real-time behavioral response of female moths to VOCs using a four-chamber olfactometer coupled with a video tracking system. Ten synthetic VOCs were selected for this study: (*S*)-(−)-perillaldehyde, (*E*)/(*Z*)-linalool oxide, (±)-limonene, linalool, (*E*)-β-caryophyllene, α/β-farnesene, (−)-α-cedrene, methyl salicylate and cumene. The effect of VOCs on egg deposition was determined using a dual-choice oviposition test, whereas perception by female antennae was verified using electroantennography (EAG). During video tracking, females responded to volatile compounds emitted by grapevine with higher antennae and ovipositor activity than to air control. (*E*)/(*Z*)-linalool oxide, cumene and (*S*)-(−)-perillaldehyde released ovipositor activity of *L. botrana*, while the latter provoked oviposition. (*R*)/(*S*)-limonene affected ovipositor activity of *E. ambiguella*, whereas none of the VOCs tested attracted for oviposition. The results suggest that females have the ability to perceive specific VOCs by the antennae but also by the ovipositor, which could attract or repel for egg deposition.

## 1. Introduction

The selection of actual host plants by herbivorous insects for reproduction is amongst others triggered by the emitted host plant bouquet, specified as volatile organic compounds (VOCs). In this regard, Lepidoptera belong to a well-studied order of insects [1], in which the European grapevine moth *L. botrana* and the European grape berry moth *E. ambiguella* are also included. They are two of the most important insect pests threatening European viticulture [2], whereas *L. botrana* has been reported as a new grape pest in the Americas: California, Chile and Argentina [3,4,5]. Both species are crepuscular [6,7] and mainly oviposit on the surface of fructiferous organs of plants, whereas several families were identified as hosts (e.g., Vitaceae, Oleaceae, Thymeleaceae and Rosaceae) [8,9,10,11,12]. Most notably, they are multivoltine on grapevine. *L. botrana* is able to complete up to four generations per year under favorable climatic conditions [13]. The first generation is considered to be exclusively anthophagous (flower-feeding) and may cause direct yield losses to a greater extent than the following carpophagous (fruit-feeding) generations [14,15]. Direct damage is caused by feeding of larvae on immature, ripening and ripe berries, which indirectly promotes an infection by the grey mold fungus *Botrytis cinerea* Persoon: Fries [16,17]. In certain cases, the direct damage favors the colonization of acetic acid bacteria and yeasts, which may cause greater incidence of sour rot in presence of *Drosophila* spp. [18,19].

Several studies have investigated the oriented flight of females and males to host plants [20,21,22,23,24], whereas less evidence has been given on short-range attraction. Especially for females, VOCs are assumed to initiate the crucial step of oviposition [25,26], which finally triggers pest infestation in vineyards. It has already been proven that *L. botrana* females have sensory structures, which allow the contact detection of physical and chemical stimuli that show no or reduced volatility after settlement on a plant [26,27]. Mainly non-porous sensilla and terminal pore sensilla, located at the legs, the ovipositor, the proboscis and the antennal tip of females are assumed to evaluate particular plants as hosts [27]. In former studies, the behavioral effect of single compounds identified in the scent bouquet of grapevine was evaluated by the flight activity of females in wind tunnel studies [28,29,30]. The perception of VOCs by female’s antennae was measured using electroantennography (EAG) [20,24], while a comparable method to evaluate the influence of VOCs on short-range attraction and oviposition activity is still missing. In particular, receptors on the ventral surface of the ovipositor are assumed to allow the detection of plant allelochemicals and/or deterrents [27,31].

Therefore, we developed a method to measure essential behavioral patterns, which reflect female’s orientation to suitable egg laying sites. The aim was to (i) quantify female short-range attraction induced by VOCs and (ii) determine the effect of volatiles on oviposition behavior. The general perceptibility of single VOCs by *E. ambiguella* and *L. botrana* female antennae was verified using EAG. To assess short-range orientation to host plants and VOCs, a four-chamber olfactometer assay was developed, which allows female behavior to be tracked, like settlement near volatile sources, flight activity and movement of the antennae or ovipositor. Especially the observation of ovipositor movement can be used as evidence for compounds, which are recognized by the ovipositor’s sensilla.

Furthermore, the results contribute to the identification of VOCs, which are responsible for females’ oviposition decisions. They can be used for the development of bait-based tools for the control of these pests.

## 2. Materials and Methods

### 2.1. Insect Rearing

Moths used in the bioassays were taken from an insect culture established at Julius Kühn-Institut, Siebeldingen, Germany. They were reared according to Markheiser et al. [32] on a semi-artificial diet. Pupae were separated by sex [12], individually transferred into 15 mL falcon tubes (VWR International GmbH, Darmstadt, Germany) and closed using a moisturized cellulose-plug. One male and one female moth (age < 24 h) were coupled 48 h before the bioassay was carried out. One hour before starting the experiments, at the beginning of dusk, couples were separated and females, which deposited >10 eggs during copulation period, were used for the study. The insect rearing was conducted under controlled climatic conditions of 14:8 h (light:dark) photoperiod, 1 h each of dusk and dawn, 23:19 ± 2 °C and 70 ± 5% relative humidity. The species *L. botrana* and *E. ambiguella* were kept separated from each other in climatic chambers ‘Fitotron type SGR233’ (Weiss Technik UK Ltd., Loughborough, UK). The day-night setback within the chambers was shifted by 8 h in advance to ensure higher oviposition activity during the studies, which preferably appears during dusk as observed by Stellwaag [7].

### 2.2. Volatile Organic Compounds (VOCs)

Potted grapevine plants, cv. ‘Regent’, were used for the validation of the behavioral studies. They were propagated by wood cuttings and cultured in a greenhouse at Julius Kühn-Institut, Siebeldingen, Germany, under controlled conditions of 23 ± 5 °C and 30 ± 10% relative humidity. A fungicide treatment against powdery mildew (*Erysiphe necator*) was conducted once a week with either Vivando (500 g/L metrafenone, BASF SE, Ludwigshafen, Germany), Talendo (200 g/L propynazide, DuPont, Wilmington, DE, USA) or Dynali (60 g/L difenoconazole +30 g/L cyflufenamide, Syngenta, Basel, Swizerland). No insecticides were applied. Fourteen days before the start of the experiment, fungicide treatments were stopped.

Synthetic VOCs examined in this study (Appendix A) were selected from literature [28,29,30,33,34]. All volatiles were identified as components of the scent of the host plants grapevine (*Vitis vinifera*) or flax-leaved daphne (*Daphne gnidium*). Except from the components mentioned in Rid et al. [34] and Cattaneo [35], VOC blends differ in their chemical composition and induce an attraction of *L. botrana* or *E. ambiguella* females in wind tunnel studies. The following ten substances were tested individually: (*S*)-(−)-perillaldehyde, (*E*)/(*Z*)-linalool oxide (furanoid), (*E*)/(*Z*)-linalool oxide (pyranoid), (±)-limonene, linalool, (*E*)-β-caryophyllene, α/β-farnesene (mixture of isomers), (−)-α-cedrene, methyl salicylate and cumene. All chemical substances except (*E*)/(*Z*)-linalool oxide (pyranoid) (Nippon Terpene Chemicals Inc., Kōbe, Japan) were purchased from Merck, Darmstadt, Germany.

### 2.3. Electroantennography (EAG)

The response of mated females’ antennae of *L. botrana* and *E. ambiguella* to volatiles was studied using EAG. Moths’ antennae were excised with fine scissors. The reference electrode of the EAG device was connected to the base of the antenna, whereas the recording electrode was connected to the tip of the antenna, while the last segment of antenna was cut off. Glass capillaries (0.58 mm I.D., Science Products, Hofheim, Germany) filled with Ringer solution (NaCl 7.5 g, KCl 0.35 g CaCl_2_ 0.21 g ad 1 L H_2_O) were used as electrodes and connected to silver wire. The analogue signal was detected with a probe (INR-II, Ockenfels Syntech^®^, Kirchzarten, Germany), captured and processed with a data acquisition controller (IDAC-2, Ockenfels Syntech^®^) and analyzed with EAG software (EAGpro, Ockenfels Syntech^®^). The air passing over the antenna was filtered using activated charcoal and humidified. To prepare the odor sources, a piece of filter paper (type 413, VWR International bvba, Belgium) was placed into the wide end of standard glass Pasteur pipettes. One µL of each test substance was pipetted onto the filter paper. The odor-loaded pipette was immediately placed in-line with the puff apparatus. For stimulation, an air puff (1 s, flow = 1.4 L/min) was passed through the pipette transporting the respective VOC to the continuous airflow (1.5 L/min) that passed over the antenna. The order of a set of puffs was as following: control (only filter paper), solvent control (only dichloromethane (DCM), Merck, Darmstadt, Germany), test substance (diluted in DCM), control, solvent control, and reference substance (diluted in DCM). This set was repeated three times per antenna while the substances were pipetted on a fresh filter paper for each set immediately before use. A refractory phase of 5 s was kept between the single puffs.

Response of the antenna was confirmed by controlling with a reference substance (*E. ambiguella*: linalool (10 µg/µL), *L. botrana*: α/β-farnesene (mixture of isomers, 10 µg/µL)). The test substances were puffed onto the antenna, and differences of the sum of the receptor potentials from individual olfactory receptor neurons [mV] were analyzed. Amplitudes statistically significantly higher than amplitudes derived from solvent control (DCM puff, eliciting mechanoreceptors and others) represent perceivable substances.

### 2.4. Four-Chamber Olfactometer Assays

The influence of volatiles on the behavior of mated *L. botrana* and *E. ambiguella* females was proven in a four-chamber olfactometer system (CADS-4CCP, Sigma Scientific LLC, Micanopy, FL, USA). It consisted of a five-port system (30 × 30 × 2.5 cm) with a removable lid out of glass (0.6 cm thickness) and wing nuts for air-tight sealing. The main body and the inlet and outlet ports were made from solid ultra-high-molecular-weight polyethylene (UHMW-PE).

**Figure 1:**
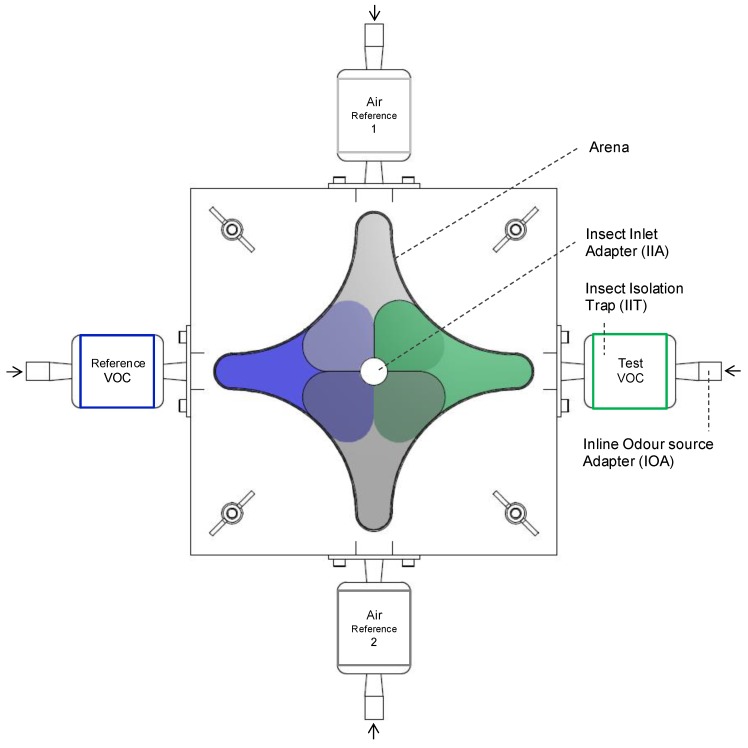
Top view of the four-chamber olfactometer. Insect arena is divided in four overlapping volatile organic compound (VOC) sectors, marked by colors. Arrows mark the connection to the clean air delivery system (CADS).

The arena, where the insects remain during inspection, was shaped like a four-pointed star (Figure 1). Each point of the star was connected to an inlet port, which enabled the insertion of either an external volatile source (plant headspace or synthetic volatile) or a reference source (clean air or solvent). Each inlet was connected to a set of borosilicate glass elements and consisted of an insect isolation trap (IIT) and a Teflon-tube adapter. The volatiles were provided via an inline odor source adaptor (IOA), which was connected to the IIT. The IIT collected insects responding to an odor source and prevented them from returning into the arena or reaching the odor source. Moths were separately introduced into the olfactometer arena via a fifth port, a bottom fed insect inlet adapter (IIA) made of borosilicate glass and equipped with a glass frit, to stop them from moving into the connected vacuum system. 

To operate the system, a four push with one pull clean air delivery system (CADS, Sigma Scientific LLC, Micanopy, FL, USA) was connected via Teflon-tubes (OD: 0.635 cm, ID: 0.396 cm) to the ports of the arena. The CADS was coupled to a compressed air source of 1.5 bar pressure. Four output (push) flowmeters controlled the flow of clean air into each port of the system and the vacuum (pull) side was connected to the IIA.

The flowmeters of the CADS were adjusted to values, which enable a uniform spread of the volatiles within the olfactometer arena. To prevent a high or low atmospheric pressure within the system, the vacuum must be equal to the sum of the four single air pressures (pvac = p1 + p2 + p3 + p4). Smoke-pens (Björnax AB, Nora, Sweden), placed inside the IITs, were used to visualize the dispersal of volatiles within the system under different airflow adjustments. The arena was divided into four identical odor sectors (color markings in Figure 1). For each pressure source at the CADS, a value of 0.2 mbar was adjusted to provide a laminar flow within the arena and hence used for the choice experiments (and 0.8 mbar for the vacuum source).

To validate the attraction of moths within the four-chamber olfactometer in general, males known to be attracted by species-specific pheromones were introduced into the system. The IIT was equipped with a pheromone source of a delta-trap (Bio-Pherotrap, Temmen GmbH, Hattersheim, Germany), specific for alluring males of *L. botrana* ((*E*,*Z*)-7,9-dodecadienyl acetate) and *E. ambiguella* ((Z)-9-dodecenyl acetate). The pheromone source was unpacked and stored in a fume cupboard 98 h before the beginning of the experiments to reduce high concentration levels within the system.

In contrast to males, the behavior of females within the system was evaluated by providing grapevine headspace. A visual healthy grapevine cluster (plant reduced to one grape cluster and four leaves 24 h before the experiment) was wrapped in an oven plastic bag (Toppits^®^, Cofresco Frischhalteprodukte GmbH & Co. KG, Minden, Germany) according to [34,36]. Cut surfaces were sealed with Parafilm^®^ to prevent an evaporation of green leaf volatiles produced by injuries. Two Teflon-tubes were, via a 50 mL Falcon tube, airproof fixed at the oven bag. One was connected to the CADS to pass air into the system and the other was connected to one port of the olfactometer system (IOA) to enable overpressure to pass off. Synthetic volatiles were provided to the females in volumes of 5 µL (1:100 dilutions in DCM; Alfa Aesar, Karlsruhe, Germany), presented on filter paper (10 × 10 mm, Schleicher & Schuell, Dassel, Germany) and inserted in the IOA. Twenty-five moths (age < 72 h) were observed per volatile source over a period of five minutes each.

Females and males were evaluated in different experimental approaches. After each trial (moth), the IOA, the arena and the glass lid of the arena were rinsed with 70% ethanol and the chemical component was renewed. At the end of the experiment, all glass elements and Teflon-tubes were cleaned with 70% ethanol and baked out at 130 °C for at least 12 h. The UHMW-PE components (arena system and IOA connecting elements) were rinsed with 70% ethanol.

### 2.5. Video Tracking System

For a uniform recording of the female’s position within the olfactometer arena, a digital camera (Basler GenICam acA1300-30um, Basler AG, Ahrensburg, Germany) was installed 1 m above the center of the arena and coupled with an EthoVision^®^XT (version 10) video tracking software (Noldus Information Technology, Wageningen, The Netherlands).

The software tracks the moth (an automatic detection) within a specific sector of the arena. Four volatile sectors were generated within the software system based on the previously observed distribution of the smoke within the arena (Figure 1). The following detection settings were adjusted: method: static subtraction; video sample rate: 25,000/s (video pixel smoothing: none; track noise reduction: off); subject is: darker (than background); dark contrast: 0–200 px; subject size: 0–750 px; subject contour: erode first, than dilate (4 px (erosion); 3 px (dilation)). During the tracking mode of static subtraction, the software calculates differences between a life image (recorded with moth) and a reference image (recorded prior to the introduction of the moth into the olfactometer system). As the tracking mode needs uniform and indirect illumination during the whole experiment, four dimmable LED lamps (Purelite 4 in 1 Crafters Magnifying lamp, Groves, Aylesbury, UK) were installed at the corners of the arena. Given that female moths are crepuscular, low intensities of illumination were necessary during the behavioral experiments. In our setup we observed 90 lx as the lowest level at which moths could be grabbed by the software system. The illumination intensity at the four exits (entry to IIT) of the arena was measured with a portable luxmeter (model 93560D, Beha Amprobe, Glottertal, Germany) and adjusted to 90 ± 2 lx, whereas the room temperature was set at 21 ± 2 °C. 

Based on the predefined trial control settings, the tracking mode was initiated automatically as soon as the moth entered one of the four volatile sectors and stopped after five minutes. Confirmed by the observed distribution of the smoke within the system, a longer period resulted in a spread of the VOC to further volatile sectors. Females were discarded if more than three minutes passed until they entered the arena. The duration (in seconds) spent in each volatile sector was calculated. Conspicuous behavior of the moth during the experiment was, after a visual registration by the operator, manually recorded by the aid of a wireless touchpad keyboard (model E2700, Rapoo Europe BV, Bergschenhoek, The Netherlands). The record of the behavior was switched on/off via a predefined button (“a” = antennae activity, “f” = flight activity and “o” = ovipositor activity) on the keyboard. The software subsequently assigned the behavior to a specific volatile sector and its duration (in seconds) and frequency for each sector was calculated.

### 2.6. Oviposition Bioassay

The effect of VOCs on oviposition was quantified using a dual choice oviposition assay in comparison to a solvent (DCM). The volatiles were offered via a dispenser system, a 2 mL Eppendorf vial filled to one third with an unscented cotton wick (Ebelin, dm-Drogerie Markt GmbH + Co. KG, Karlsruhe, Germany). The cotton wick was loaded with the test substance (1:100 dilution in DCM; Alfa Aesar, Karlsruhe, Germany) and covered by 150 µL paraffin oil (Sigma-Aldrich Chemie GmbH, München, Germany) to enable a slow release of the volatile. 

Each volatile was checked in two volumes (vial either loaded with 5 µL or 10 µL). Each dispenser was fixed with two-sided tape at the lid inside a transparent polypropylene cup (100 mL, Kastelplast GmbH, Mainz-Mombach, Germany) to offer an artificial surface for oviposition. Following [25], the cups were perforated with a needle (60 holes per cup, Ø = 1.1 mm), so volatiles could evaporate. The two prepared dispenser cups (test substance in DCM and DCM) were placed in the middle of a gauze cage (60 × 40 × 40 cm, The Caterpillar Castle, Live Monarch Foundation, Boca Raton, FL, USA), 20 cm apart from each other. 

Analogous to Rid et al. [34], a grapevine cluster cv. ‘Regent’ (BBCH 77), containing 10 berries of *V. vinifera* ‘Regent’, was used as positive control to validate the experimental setup. As negative control, in order to exclude any influence of the solvent or paraffin oil on oviposition of *E. ambiguella* or *L. botrana*, the solvent was checked against a cup containing a vial filled exclusively with a cotton wick. Four couples (sexed < 48 h) were introduced into one cage to enable adequate egg amounts. Eight repetitive cages were provided for each volatile, volume and species, changing the position of the solvent and the VOC between cages in order to avoid a position effect on egg deposition. The cages were kept in climatic chambers ‘Fitotron type SGR233’ (Weiss Technik UK Ltd., Loughborough, UK) at 23:19 ± 2 °C, 70 ± 10% relative humidity and a 14:8 h photoperiod with either 1 h dusk or dawn. Moth species were kept in separated chambers. The experiment was stopped after 72 h by removing the couples from the cage and counting the eggs deposited outside the cups. After each experiment, the climatic chambers were warmed at 60 °C for at least 3 h to enable remained odors to volatilize.

### 2.7. Statistical Analyses

All data were analyzed using the software R—version 3.6.0—‘Planting of a Tree’ [37]. Statistical differences of the response of the antennae (EAG experiments) by different chemical compounds compared to the respective DCM control were achieved using linear mixed models (LMs) for each species using package ‘*lme4*’ [38] with ‘substance’ as fixed, ‘antenna-ID’ as random factor and ‘compound-DCM pair’ nested in antenna-ID. Post hoc comparisons between the responses were obtained from estimated marginal means (EMM) using function ‘*emmeans*’ [39]. 

For olfactometer assays, the frequency per moth (FPM) and duration (in seconds) per moth (DPM) spent in each volatile sector were recorded in total and during conspicuous behavioral traits (flight-, antennae- and/or ovipositor-activity). Statistically significant differences between the four volatile sectors in the arena were calculated using generalized linear models (GLMs) with ‘behavior’, ‘moth species’ and ‘volatile source’ as fixed factors. Statistical models were compared using AICs (Aikaike information criterions) and simplified by removing non-significant factors with the function ‘drop1’ and F-test. Time measurements and frequencies were transformed by log(y + 1). The experiment was set as valid, if total settlement duration in both air references was not statistically different. In cases of a settlement of specimens in two overlapping sectors, the time and frequency were counted to both sectors. Post hoc comparisons between durations (alternative frequencies) spent by the species in single volatile sectors were generated using EMMs and Tukey’s method for *p*-value adjustment. Significance level was set at *p* < 0.05.

Relative attractiveness induced by volatiles in the oviposition assay was calculated using the oviposition discrimination index (ODI) mentioned in [40]: ODI = [(number of eggs on cup A (test VOC) − number of eggs on cup B (solvent))/total number of eggs] × 100%. The value varies from −100 (negative effect on oviposition) to +100% (positive effect on oviposition). Statistical analysis was carried out by applying non-parametric Wilcoxon signed rank test for paired data sets (egg counts). 

Box-whisker plots were created using R-package ‘*ggplot2*’ [41], whereby lines represent the median, dots the mean, boxes the interquartile range (IQR), whiskers 1.5 × IQR and dots outside boxes the outliers. Heat maps were plotted using EthoVision^®^XT (version 10) video tracking software (Noldus Information Technology, Wageningen, The Netherlands).

## 3. Results

### 3.1. Perception of VOCs by EAG

The absolute response [mV] differed between antennae and between sets of antennae due to their viability and lifetime. For that reason, the antenna ID was used as random factor and the response to the test substance was compared to its respective DCM control. The mean absolute response to ten chemical compounds (10 µg) was calculated both for *E. ambiguella and L. botrana* (Table 1) and compared to the DCM control. All 10 test substances can be perceived by the antenna of both moth species.

### 3.2. Responsiveness of Moths in the Olfactometer

#### 3.2.1. Males

Pheromones were used to quantify the general attraction of moths within the olfactometer system. Males of *E. ambiguella* (n = 22 of 25) and *L. botrana* (n = 23 of 25) almost entirely entered the olfactometer system within a period of three minutes. During the observation period of five minutes, flight activity was observed in 27.3% of *E. ambiguella* and in 60.9% of *L. botrana* specimens. The frequency and duration spent by males in each volatile sector was analyzed (Table 2). Due to the frequent flight interruption, the males’ antenna activity could not be recorded during the tracking mode.

**Figure 2:**
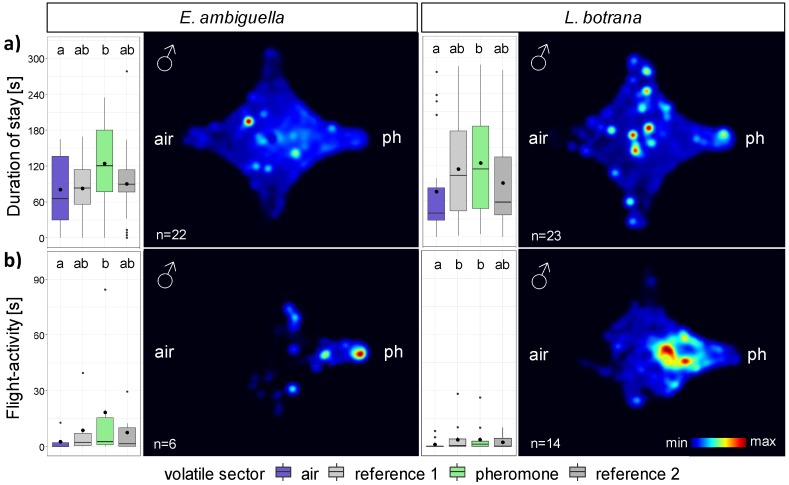
Box-whisker plots and merged heat maps visualizing the duration of (**a**) stay and (**b**) flight activity of (left) *E. ambiguella* and (right) *L. botrana* males within the four-chamber olfactometer arena after introduction of a pheromone source (ph). Different letters indicate statistical differences between volatile sectors according to generalized linear model (GLM) and post hoc comparisons using estimated marginal means (EMMs) (*p* < 0.05).

The moth species had no significant influence on the duration of stay within single sectors (GLM: F_1,254_ = 0.93; *p* = 0.33), hence data of *E. ambiguella* and *L. botrana* were merged for further analyses. The duration of males’ flight activity in single sectors was short (4.6 ± 1.3 s, n = 80) if compared to the duration of stay in the single sectors of the total recording time (97.7 ± 5.3 s, n = 180) (GLM: F_1,254_ = 419.82; *p* < 0.001). The volatile sector had a statistically significant influence on the settlement duration of males (GLM: F_3,254_ = 3.41; *p* = 0.016, Figure 2). 

Independent from the observed behavior (stay (n = 45) or flight activity (n = 20)), the cumulative time spent by *E. ambiguella* and *L. botrana* in the pheromone sector was statistically (z_4_ = 3.14, *p* < 0.01) longer (stay: 124.0 ± 11.2 s; flight activity: 8.1 ± 4.3 s) than in the air sector (stay: 78.1 ± 10.2 s, flight activity: 1.5 ± 0.8 s).

The moth species influenced the frequency of volatile sector entries (GLM: F_1,254_ = 13.03; *p* < 0.001), with males of *E. ambiguella* switching more frequently between volatile sectors than males of *L. botrana* (z_2_ = 3.61, *p* < 0.001). In contrast, the volatile source had no significant influence on the frequency of entering a volatile sector (GLM: F_3,254_ = 0.96; *p* = 0.41).

#### 3.2.2. Females

The behavior of mated females near volatile compounds emitted by grapevine (*V. vinifera* ‘Regent’; BBCH 77) served as model for female moths, which are in search of oviposition sites. Activities of the female’s antennae were characterized by a pivoting up and down movement of the antennae while standing still. The ovipositor activity was recognizable by palpation of the olfactometer surface with the ovipositor, whereby the abdomen was swinging back and forth. Flight activity of females was rare, so that this behavior was not recorded in our experiments (Table 3).

The duration spent by females in a volatile sector of the olfactometer arena (Table 3) was influenced by the factors behavior (GLM: F_1,333_ = 121.38; *p* < 0.001), moth species (GLM: F_1,333_ = 8.46; *p* < 0.01) and volatile source (GLM: F_3,333_ = 4.50; *p* < 0.01). Females of *E. ambiguella* spent significantly less time (84.6 ± 1.3 s) within the arena than females of *L. botrana* (95.6 ± 9.0 s) (z_2_ = −2.91, *p* < 0.001), which is a consequence of moths moving back to the IIA. Considering the sum of DPM over all four volatile sectors, both species had a significantly shorter duration (z_3_ = −5.29, *p* < 0.001) of antennae activity (*E. ambiguella*: 1.0 ± 0.3 s; *L. botrana*: 3.3 ± 0.8 s) than ovipositor activity (*E. ambiguella*: 17.2 ± 9.5 s; *L. botrana*: 22.1 ± 4.0 s). 

**Figure 3:**
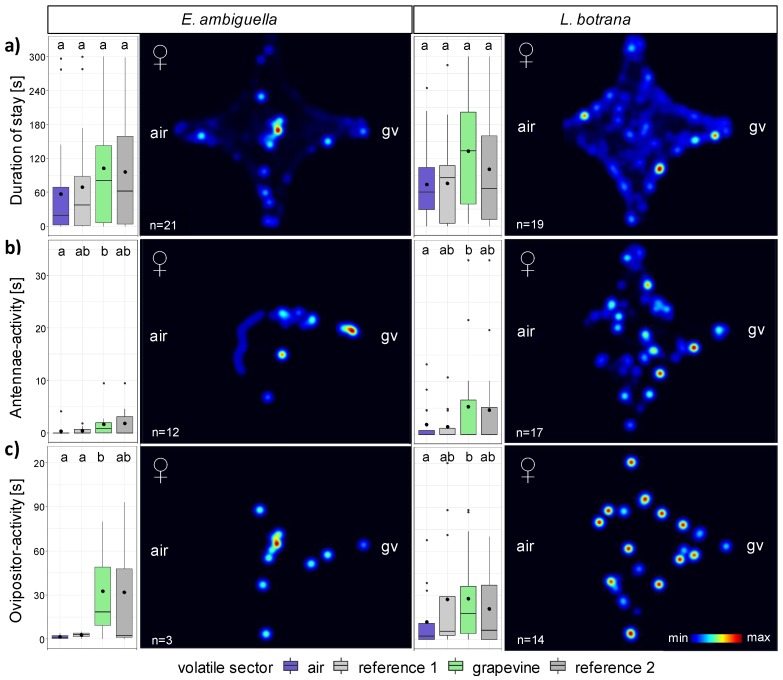
Box-whisker plots and merged heat maps visualizing the duration of (**a**) stay (**b**) antennae activity and (**c**) ovipositor activity of (left) *E. ambiguella* and (right) *L. botrana* females within the four-chamber olfactometer arena after introduction of a grapevine headspace (gv). Different letters indicate statistical differences between volatile sectors according to generalized linear model (GLM) and post hoc comparisons using estimated marginal means (EMMs) (*p* < 0.05).

The duration of stay within single volatile sectors could not be explained by the volatile source, neither for *E. ambiguella* (GLM: F_3,76_ = 2.17; *p* = 0.10, Figure 3a left) nor for *L. botrana* (GLM: F_3,72_ = 1.64; *p* = 0.19, Figure 3a right). For both moth species, the duration of antennae (Figure 3b) and ovipositor activity (Figure 3c) was triggered by the volatile source. Antennae (z_4_ = −2.01, *p* < 0.05) and ovipositor activities (z_4_ = −3.44, *p* = 0.01) were statistically shorter in the air sector than in the grapevine sector. 

Differences in the frequency of volatile sector entries (Table 3) were a result of the behavior (GLM: F_2,333_ = 91.30; *p* < 0.001) rather than the insect species (GLM: F_1,333_ = 0.22; *p* = 0.64) or volatile source (GLM: F_3,333_ = 1.75; *p* = 0.15).

### 3.3. Short-Range Attraction of Females by VOCs

Due to the fact that females previously did not enter the grapevine sector more frequently than the air sector, we focused in the further studies on the duration spent by the females in the single volatile sectors (full dataset in Table S1).

**Figure 4:**
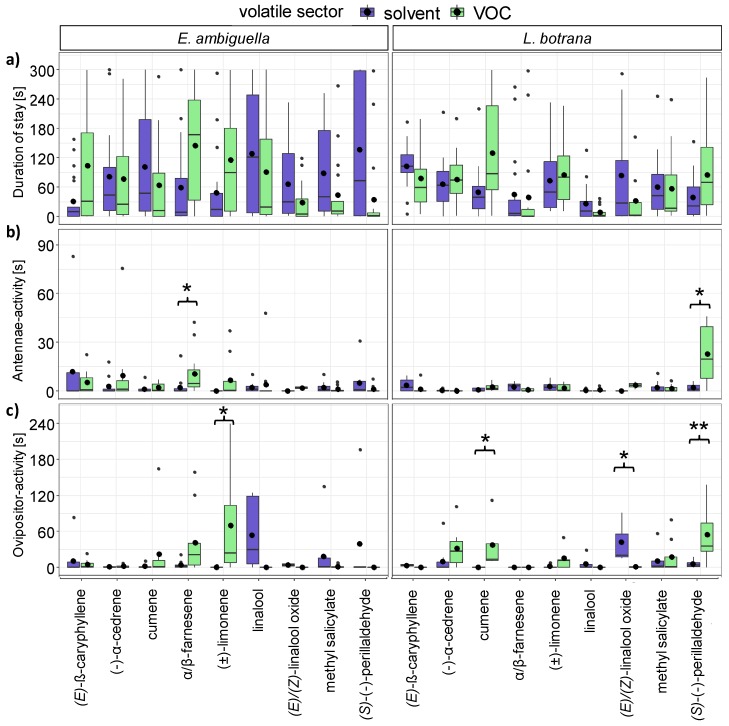
Box-whisker plots visualizing the duration of (**a**) stay, (**b**) antennae activity and (**c**) ovipositor activity of (left) *E. ambiguella* and (right) *L. botrana* females within the four-chamber olfactometer arena after introduction of different volatile organic compounds (VOCs). Asterisks indicate statistical differences between VOC and solvent control according to generalized linear model (GLM) and post hoc comparisons using estimated marginal means (EMMs) (* *p* < 0.05; ** *p* < 0.01, n = 25).

Although behavior was a factor explaining differences in the duration in all VOCs tested in this study (statistics in Table S2), there were no statistically significant differences between the duration of antennae and ovipositor activity (data not shown). None of the VOCs ((±)-limonene and (*E*)/(*Z*)-linalool oxide (pyranoide/furanoide) tested as isomere mixture 1:1) had a statistically significant influence on duration of stay within single volatile sectors during the total observation period (GLM; *p* > 0.05; Figure 4a).

The volatiles α/β-farnesene (mixture of isomers) and (*±*)-limonene influenced the behavior of *E. ambiguella* significantly (Table S2). The antennae activity of *E. ambiguella* was significantly higher (z_4_ = 3.1; *p* < 0.05, Figure 4b) in the farnesene sector than in the sector of the solvent control. Further, (*±*)-limonene had a positive effect on the ovipositor activity, which was higher in the volatile sector (z_4_ = 2.63; *p* < 0.05, Figure 4c) than in the solvent control.

The behavior of *L. botrana* was influenced by the compounds cumene, (*E*)/(*Z*)-linalool oxide (pyranoide/furanoide) and (*S*)-(−)-perillaldehyde (Table S2). In comparison to the solvent control, the ovipositor activity was significantly higher in the volatile sector of cumene (z_4_ = 2.72; *p* < 0.05, Figure 4c) as well as (*S*)-(−)-perillaldehyde (z_4_ = 3.35; *p* < 0.01, Figure 4c) and significantly lower (z_4_ = -2.62; *p* < 0.05, Figure 4c) in the volatile sector of (*E*)/(*Z*)-linalool oxide (pyranoide/furanoide). The compound (*S*)-(−)-perillaldehyde additionally promoted the antennae activity of *L. botrana* in comparison to DCM significantly (z_4_ = 2.16; *p* < 0.05, Figure 4b).

### 3.4. Oviposition Induced by VOCs

**Figure 5:**
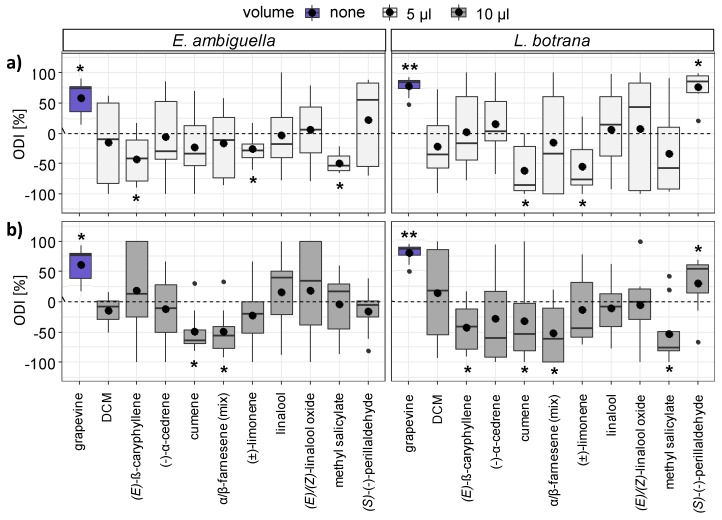
Oviposition preferences of (left) *E. ambiguella* and (right) *L. botrana* induced by volatile organic compounds (VOCs) (1:100 dilution in dichloromethane (DCM)) in a volume of (**a**) 5 µL and (**b**) 10 µL (grapevine = positive control, DCM = negative control). Preferences expressed by oviposition discrimination indices (ODI) (negative ODI = repellent; positive ODI = attractant). Asterisks indicate statistical differences between solvent control (DCM) and VOC according to Wilcoxon signed rank test (* *p* < 0.05, ** *p* < 0.01; n = 8).

The influence of VOCs (1:100 dilutions in DCM) on oviposition behavior of *E. ambiguella* and *L. botrana* was dose dependent (Figure 5). None of the compounds tested in our studies attracted *E. ambiguella* females for oviposition (Figure 5, left) when compared to the solvent control, whereas dispenser systems equipped with (*S*)(−)-perillaldehyde significantly (Wilcoxon signed rank test, *p* < 0.05) attracted females of *L. botrana* for oviposition in a volume of 5 µL (Figure 5a, right).

The compounds (*E*)-β-caryophyllene, (±)-limonene and methyl salicylate (5 µL) were significant in reducing the egg deposition of *E. ambiguella*, whereas a comparable effect was observed in the case of cumene and (±)-limonene (5 µL) for *L. botrana* (Figure 5a).

Increasing the volume to 10 µL (Figure 5b) resulted in none of the VOCs tested having a positive effect on oviposition. Dispenser systems were either avoided or no longer discriminable to the solvent. Cups equipped with the compounds cumene and α/β-farnesene in a volume of 10 µL were decreasing the egg deposition of *E. ambiguella*, whereas (±)-limonene, (*E*)-β-caryophyllene, cumene, α/β-farnesene and methyl salicylate repelled oviposition of *L. botrana*.

## 4. Discussion

Grapevine moths do not require the stimulus of a natural plant to accept surfaces for oviposition [25,32,40]. *L. botrana* is essentially nocturnal and mating flight is initiated during night, while oviposition starts with sunset and lasts until night. In contrast, *E. ambiguella* is flight active during dawn and egg laying was observed at noon until night [7,42]. Hence, any visual component in host-seeking behavior, which is known to play a key role in some insects that are active by day [43] is assumed to be less significant, at least for *L. botrana* [25,32]. Therefore, response to volatile plant odors and fruit surface condition are probably the most likely mechanisms for Lepidoptera species like *E. ambiguella* and *L. botrana* for locating suitable host plants for reproduction [21,24,26,27,30,44,45,46]. Plant odors enhance the attraction to pheromones in many species, having the potential as low-cost attractants in traps [47,48]. The synthetic VOCs selected for this study were confirmed to be perceivable by the antennae of *E. ambiguella* and *L. botrana* females in EAG experiments and possibly attract female moths for reproduction. Some of them were derived from plants other than grapevine. A single compound, (*S*)-(−)-perillaldehyde, a volatile emitted by the non-host plant *Perilla frutescens* [33], was found to promote oviposition of *L. botrana*. Further VOCs may trigger the female’s attraction for reproduction in a similar way.

Oviposition response to VOCs is often assessed over longer periods of time and relies on passive dispenser systems [25,40,44,49,50], which possibly allow a rapid degradation of the chemical compounds [48]. As a consequence, it is difficult to estimate concentration levels, which operate as attractant or repellent for egg deposition. 

Hence, this study aimed at developing a four-chamber olfactometer assay to measure the real-time behavioral responses of adult *E. ambiguella* and *L. botrana* to VOCs over a short range. Within a four-chamber olfactometer, the air flow builds up sectors of different odor levels, visualized by the distribution of the smoke emitted by the pens. Thus, insects were allowed to select within the arena sectors of preferred odor concentrations, an advantage in comparison to a Y-olfactometer, where moths can only respond to one odor level. Furthermore, it is possible to quantify further behavioral patterns such as sensory probing of the surface by the ovipositor, egg deposition, antennae and flight movements or female calling behavior. This enables the role of the respective substances in the behavior of the grapevine moths to be analyzed.

In the case of males, we observed increased flight activity and duration of stay near the pheromone sector within the olfactometer arena for both species and conclude that the system is suitable to quantify volatile compounds. Based on previous results, which confirmed the attraction of females of *L. botrana* to grape odors in wind tunnels and/or Y-olfactometer experiments [21,24,30,34,51], both species were exposed to the headspace of *V. vinifera* ‘Regent’. Independently of moth species, we observed increased ovipositor and antennae activity in the arena’s sector which was enriched with grapevine odor. Regarding the duration of stay, the lack of response of females could be a consequence of the plant material tested (e.g., grape variety, amount of plant material and the release rate of plant volatiles) [52] or the lack of required supplementary signals necessary in locating host plants [25]. Possibly, the settlement position of the moth within the arena system, which is known to trigger oviposition [32], has a higher priority in the stimulus cascade relevant for oviposition than olfactory stimuli. The behavior of females, which try to locate suitable egg-laying sites, is reviewed by Galet [53] citing various authors. According to this review, females of both species fly agitatedly between grape clusters and lay eggs on different widespread grapes. We assume that this behavior is reflected by females in the olfactometer system, resulting in duration of stay being evenly distributed over volatile sectors, while orientation to directions (antennae activity) and tasting (ovipositor activity) is influenced by volatiles.

The positive effect of (*S*)-(−)-perillaldehyde on egg deposition and ovipositor activity could be confirmed for *L. botrana* in our studies. Ovipositor activity was also higher in the volatile sector than in the solvent control of the compounds (±)-limonene (*E. ambiguella*), (*E*)/(*Z*)-linalool oxide (pyranoide/furanoide) and cumene (*L. botrana*), whereas none of these compounds operated as attractant for oviposition. This highlights that increased egg deposition is not necessarily a result of increased ovipositor activity. It may rather reflect a gustatory perception of the VOC by the ovipositor receptors as hypothesized by Maher and Thiéry [27]. Cumene, a compound recently identified in the bouquet of grapevine [34], was perceived by the female moth’s antennae with lower electrical responses (*E. ambiguella*: −0.67 ± 0.35 mV; *L. botrana*: −0.37 ± 0.10 mV) when compared to α/β-farnesene (*E. ambiguella*: −1.45 ± 0.61 mV; *L. botrana*: −1.12 ± 0.40 mV), while *L. botrana* females showed increased ovipositor activity within the volatile sector of cumene in the olfactometer system. We may assume a higher detection with organs other than antennae resulting in a behavioral output.

None of the VOCs tested in this study provoked an increased duration of stay within the volatile sector in comparison to the solvent control. This may be a result of the VOC dose or the kind of compound tested. In the olfactometer experiments, the dose was adjusted to the same as in our EAG experiments (10 µg/µL), which conforms to other studies with grapevine moths [21,22,24]. Deviating VOC doses may result in changes of the behavioral response of females as observed for *L. botrana* in Y-olfactometer experiments [54] or oviposition tests in this study.

Furthermore, moth species such as *L. botrana* possibly avoid artificial and natural oviposition sites treated with particular VOCs as, e.g., shown by Thiéry et al. [55] for the substance methanol or Silva et al. [54] for essential oils of the non-host plant *Schinus molle* L. In this study, some compounds (methyl salicylate, α/β-farnesene and (*E*)-β-caryophyllene) had a negative effect on egg deposition while presenting no increased ovipositor activity in comparison to solvent. These compounds are probably to a greater extent perceived by antennae rather than other extremities of females as suggested by Maher and Thiéry [27].

The compound methyl salicylate, derived from salicylic acid, is released in larger amounts by many plants after damage, infection or abiotic stress [56,57,58,59]. In some cases, this VOC acts as plant chemical defense to attract natural enemies [59,60,61]. However, such plants may be less preferred by gravid females for oviposition due to the lack of berry persistence and/or the nutritional conditions necessary for offspring development. For instance, the odor of *Botrytis cinerea*-inoculated fruits reduced oviposition of *L. botrana* [62]. This may explain the repellent effect on oviposition in this study. Furthermore, Ulland et al. [63] found this compound to reduce the oviposition in the cabbage moth *Mamestra brassicae*. 

The isomer mixture of α/β-farnesene had no influence on the attraction of both species. This may be a result of the dose and/or the kind of isomere mixture tested. It may contain sesquiterpenes, (*E*)-β-farnesene, (*Z*,*Z*)-α-farnesene, (*Z*,*E*)-α-farnesene and bisabolene. Two of the four stereoisomers of α-farnesene, (*E*,*E*)-α-farnesene and (*Z*,*E*)-α-farnesene, attracted larvae of the codling moth *C. pomonella*, while (*E*)- and (*Z*)-isomers of β-farnesene had no effect on larvae under controlled conditions [64,65,66]. The isomer (*E*,*E*)-α-farnesene was additionally found to promote oviposition of *C. pomonella* [67]. Furthermore, *C. pomonella* responded with walking in Y-olfactometer assays at a higher rate to 0.01 µg of a (*E*,*E*)- and (*Z*,*E*)-α-farnesene isomer mixture than to other doses tested (0.001, 0.1, 1, 10 µg) and solvent control [68]. Ongoing studies should therefore rely on testing a variety of VOC doses and all isomers. 

Future studies aim to decode essential VOCs affecting host plant acceptance for reproduction of grapevine moths. The method developed in this study could be used to validate the suitability of VOCs for short-range attraction and measure behavioral patterns related to oviposition. This will finally support the development of a so-called ‘m-ovi-card’, a specific egg-monitoring tool, which aims at reflecting critical threshold levels of pest infestations in vineyards to prohibit immoderate insecticide applications [26,32,34]. Therefore, a combination of attractive VOCs and an artificial surface that fulfills the visual and tactile requirements necessary for grape moth females to accept surfaces for oviposition is still under investigation.

## 5. Conclusions

This study indicates that the short-range attraction of female grapevine moths *E. ambiguella* and *L. botrana* can be triggered by VOCs. We assume that VOCs emitted by grapevine are essential for short-range orientation due to the fact that the female’s antennae activity was longer in the grapevine than in the air sector. The four-chamber olfactometer system enables important behavioral patterns to be tracked, like ovipositor activity, which are often disregarded in the evaluation of physical and chemical stimuli for oviposition. Increased durations of ovipositor activities suggest that females have the ability to recognize specific VOCs by the ovipositor, which could attract or repel females for egg deposition. 

## Figures and Tables

**Table 1:** Average absolute responses of antennae of both *E. ambiguella* and *L. botrana* females to ten chemical compounds and to air and solvent control (dichloromethane (DCM)) obtained using EAG experiments.

Compound	*E. ambiguella*				*L. botrana*			
	n	Response [mV] ± SD	*p*-Value ^1^		n	Response [mV] ± SD	*p*-Value ^1^	
(*E*)-β-caryophyllene	3 × 5	−1.14 ± 0.22	9.30 × 10^−12^	***	3 × 5	−0.63 ± 0.23	3.30 × 10^−9^	***
(−)-α-cedrene	3 × 5	−0.63 ± 0.22	2.35 × 10^−7^	***	3 × 5	−0.41 ± 0.18	1.13 × 10^−3^	**
cumene	3 × 5	−0.67 ± 0.35	1.39 × 10^−8^	***	3 × 5	−0.37 ± 0.10	5.85 × 10^−8^	***
α/β-farnesene	3 × 5	−1.45 ± 0.61	9.94 × 10^−7^	***	3 × 5	−1.12 ± 0.40	7.58 × 10^−10^	***
(+)-limonene	3 × 5	−0.82 ± 0.39	1.79 × 10^−7^	***	3 × 5	−0.58 ± 0.24	1.69 × 10^−8^	***
(−)-limonene	3 × 5	−0.96 ± 0.35	1.25 × 10^−7^	***	3 × 5	−0.82 ± 0.26	6.47 × 10^−8^	***
linalool	3 × 5	−1.88 ± 0.46	8.82 × 10^−14^	***	3 × 5	−1.29 ± 0.41	1.35 × 10^−10^	***
(*E*)/(*Z*)-linalool oxide (furanoid)	3 × 5	−1.40 ± 0.51	2.77 × 10^−11^	***	3 × 5	−0.69 ± 0.22	1.89 × 10^−7^	***
(*E*)/(*Z*)-linalool oxide (pyranoid)	3 × 5	−0.98 ± 0.67	1.68 × 10^−5^	***	3 × 5	−0.80 ± 0.26	1.62 × 10^−10^	***
methyl salicylate	3 × 5	−1.10 ± 0.43	1.07 × 10^−8^	***	3 × 5	−1.06 ±0.46	2.39 × 10^−7^	***
DCM	3 × 50	−0.44 ± 0.26			3 × 50	−0.36 ± 0.18		
air	3 × 50	0.48 ± 0.34			3 × 50	0.38 ± 0.22		

^1^ Statistical differences between the response to the compound and to DCM control by linear mixed model (LM), post hoc estimated marginal mean (EMM) (*** *p* < 0.001; ** *p* < 0.01).

**Table 2:** Frequency per moth (FPM) and duration per moth (DPM) spent by males of *E. ambiguella* (EA) and *L. botrana* (LB) in the four volatile sectors of the olfactometer system during stay (total observation period of 300 s) and flight activity after introducing a pheromone source (pher).

Species	Behavior	n	FPM in Sector (Mean ± SE) [n]				DPM in Sector (Mean ± SE) [s]			
			Air	Reference 1 (Air)	Pher	Reference 2 (Air)	Air	Reference 1 (Air)	Pher	Reference 2 (Air)
EA	stay	22	41.4 ± 6.3	55.6 ± 9.9	47.3 ± 6.9	58.2 ± 10.4	80.5 ± 12.2	82.8 ± 9.5	124.2 ± 14.9	90.8 ± 13.0
	flight	6	5.2 ± 3.6	9.6 ± 6.7	7.8 ± 4.9	10.7 ± 7.6	2.6 ± 2.1	8.8 ± 6.3	18.3 ± 13.6	7.5 ± 4.8
LB	stay	23	30.7 ± 7.0	29.0 ± 4.2	31.4 ± 4.7	37.6 ± 7.4	75.9 ± 16.6	113.2 ± 17.0	123.8 ± 16.9	90.2 ± 16.2
	flight	14	0.5 ± 0.4	1.6 ± 0.7	1.4 ± 0.5	2.0 ± 1.1	1.0 ± 0.7	3.7 ± 2.0	3.7 ± 1.9	2.3 ± 0.9

**Table 3:** Frequency per moth (FPM) and duration per moth (DPM) spent by females of *E. ambiguella* (EA) and *L. botrana* (LB) in the four volatile sectors of the olfactometer system during stay (total observation period of 300 s) and specific behaviors (antennae and ovipositor activity) after introducing a grapevine headspace (grape).

Species	Behavior	n	FPM in Sector (Mean ± SE) [n]				DPM in Sector (Mean ± SE) [s]			
			Air	Reference 1 (Air)	Grape	Reference 2 (Air)	Air	Reference 1 (Air)	Grape	Reference 2 (Air)
EA	stay	21	32.8 ± 9.8	25.3 ± 9.1	35.5 ± 10.5	25.8 ± 7.4	42.7 ± 19.1	45.5 ± 19.0	82.3 ± 25.4	72.6 ± 22.7
	antennae	12	0.3 ± 0.3	0.6 ± 0.3	1.6 ± 0.5	1.0 ± 0.4	0.3 ± 0.3	0.4 ± 0.2	1.7 ± 0.8	1.8 ± 0.9
	ovipositor	3	0.7 ± 0.3	1.0 ± 0.6	4.7 ± 2.9	1.3 ± 0.9	1.6 ± 1.2	2.7 ± 1.5	32.7 ± 24.1	31.8 ± 30.6
LB	stay	19	11.9 ± 2.5	11.2 ± 2.4	28.0 ± 12.1	11.5 ± 3.0	73.8 ± 15.4	75.4 ± 17.6	132.6 ± 22.1	100.5 ± 21.8
	antennae	17	0.7 ± 0.3	0.6 ± 0.2	1.4 ± 0.5	1.1 ± 0.5	1.9 ± 0.9	1.5 ± 0.7	5.3 ± 2.2	4.6 ± 2.2
	ovipositor	14	2.4 ± 0.7	3.1 ± 0.8	3.2 ± 0.8	2.2 ± 0.6	12.1 ± 5.4	27.5 ± 10.2	27.9 ± 8.6	21.0 ± 7.5

## Supplementary Materials

The following are available online at https://www.mdpi.com/2075-4450/11/1/45/s1, Table S1: Frequency (FPM) and duration (DPM) spent by *E. ambiguella* and *L. botrana* females in each of the four volatile sectors of the olfactometer system during specific behaviors (stay, antenna and/or ovipositor activity) after introducing a volatile organic compound (VOC) and a solvent (DCM) source in opposite sectors, Table S2: Summary of statistical parameters explaining the duration spent by *E. ambiguella* or *L. botrana* within the olfactometer arena.

## Appendix A

**Table A1:** Evaluated volatile organic compounds selected from synthetic volatile blends attracting females of *L. botrana* in wind tunnel studies [28,29,30] and/or identification from grapevine headspace [34].

Compound	Tasin et al., 2007		Anfora et al., 2009	Tasin et al., 2010		Rid et al., 2019
	L *	B *	PS 33 *	GM *	DM *	Grapevine *
**Monoterpenes**						
(*E*)/(*Z*)-linalool oxide (furanoid)	◦	●	◦	●	●	●
(*E*)/(*Z*)-linalool oxide (pyranoid)	◦	◦	◦	◦	●	◦
(±)-limonene	◦	◦	●	◦	◦	●
linalool	◦	●	●	●	●	●
**Sesquiterpenes**						
(*E*)-β-caryophyllene	●	●	●	●	●	●
(*E*)-β-farnesene	●	◦	◦	●	◦	●
(*E*,*E*)-α-farnesene	◦	●	●	●	●	●
(−)-α-cedrene	◦	◦	◦	◦	◦	●
**Benzenoids**						
methyl salicylate	◦	●	●	●	●	●
cumene	◦	◦	◦	◦	◦	●

● Compound present; ◦ Compound absent. * Name of the volatile mixture used in the literature cited.
