# Supplementary material for: Tracking Short-Range Attraction and Oviposition of European Grapevine Moths Affected by Volatile Organic Compounds in a Four-Chamber Olfactometer
Source: Insects. 2020 Jan 8;11(1):45. doi: 10.3390/insects11010045 (PMC7022303; doi:10.3390/insects11010045)
Supplement: Supplementary file 1 [file insects-11-00045-s001.docx]

Table S1. Frequency (FPM) and duration (DPM) spent by *E. ambiguella* and *L. botrana* females in each of the four volatile sectors of the olfactometer system during specific behaviors (stay, antenna- and/or ovipositor-activity) after introducing a volatile organic compound (VOC) and a solvent (DCM) source in opposite sectors.

| **female species** | **VOC** | **behavior** | **n** | **FPM in sector (mean ± SE) [n]** | | | | **DPM in sector (mean ± SE) [s]** | | | |
| --- | --- | --- | --- | --- | --- | --- | --- | --- | --- | --- | --- |
|  |  |  |  | DCM | reference 1 (air) | VOC | reference 2 (air) | DCM | reference 1 (air) | VOC | reference 2 (air) |
| *E. ambiguella* | *(E)*-β-caryo-  phyllene | stay | 21 | 15.5 ± 11 | 13.6 ± 9.4 | 5.2 ± 2.1 | 10.3 ± 3.8 | 30.8 ± 10.6 | 60.8 ± 20.5 | 103.8 ± 25.9 | 105.7 ± 25.1 |
|  |  | antennae | 9 | 1.9 ± 1.2 | 1.6 ± 0.9 | 1.4 ± 0.8 | 0.2 ± 0.2 | 11.9 ± 9 | 5.7 ± 3.1 | 5.1 ± 2.6 | 0.1 ± 0.1 |
|  |  | ovipositor | 10 | 5 ± 4.2 | 1.4 ± 0.8 | 1.8 ± 0.8 | 0.2 ± 0.2 | 10.9 ± 8.2 | 5.1 ± 2.8 | 4.8 ± 2.3 | 0.1 ± 0.1 |
|  | (-)-α-cedrene | stay | 17 | 6 ± 1.2 | 4.4 ± 0.9 | 7.5 ± 2.3 | 7 ± 2.3 | 80.8 ± 22.8 | 97.3 ± 22.7 | 76.4 ± 24.2 | 83.7 ± 21.1 |
|  |  | antennae | 11 | 1.2 ± 0.6 | 1.6 ± 0.6 | 2.5 ± 1.1 | 2 ± 0.8 | 2.7 ± 1.7 | 9.4 ± 5.7 | 9.6 ± 6.7 | 4.5 ± 2.1 |
|  |  | ovipositor | 4 | 0.8 ± 0.2 | 0.8 ± 0.5 | 2 ± 1.7 | 1.8 ± 1.4 | 0.6 ± 0.4 | 34.5 ± 34.2 | 1.8 ± 1.6 | 2.4 ± 1.6 |
|  | cumene | stay | 20 | 5.3 ± 1.3 | 3.5 ± 1.1 | 7.7 ± 3.5 | 16.2 ± 12.8 | 101.2 ± 25.4 | 127.4 ± 27.9 | 63.8 ± 19.6 | 98.1 ± 24.7 |
|  |  | antennae | 12 | 0.5 ± 0.2 | 1 ± 0.3 | 0.9 ± 0.3 | 0.8 ± 0.4 | 1.1 ± 0.7 | 1.8 ± 0.8 | 2 ± 0.8 | 3.9 ± 2.4 |
|  |  | ovipositor | 9 | 0.9 ± 0.5 | 1.6 ± 0.4 | 1.7 ± 1 | 1.1 ± 0.6 | 1.5 ± 1.1 | 14.4 ± 9.7 | 22.2 ± 17.9 | 6.5 ± 4.9 |
|  | α/β-farnesene (mixture of isomers) | stay | 19 | 4.5 ± 1.9 | 2.7 ± 0.8 | 2.9 ± 0.9 | 2.6 ± 0.7 | 59.4 ± 19.9 | 144.1 ± 28.7 | 145.4 ± 26 | 74.3 ± 26.4 |
|  |  | antennae | 14 | 1.1 ± 0.6 | 3.1 ± 0.8 | 4.1 ± 1.3 | 2.1 ± 1.4 | 2.2 ± 1.5 | 9.4 ± 3.3 | 10.4 ± 3.5 | 3.7 ± 2.9 |
|  |  | ovipositor | 10 | 1.2 ± 0.4 | 2.1 ± 0.4 | 2.6 ± 0.7 | 1.4 ± 0.9 | 4 ± 2.1 | 19.2 ± 6.6 | 41 ± 17.3 | 21.3 ± 14.6 |
|  | *(±)*-limonene | stay | 17 | 4.7 ± 1.7 | 102.3 ± 97.7 | 30.3 ± 26.7 | 2.6 ± 0.7 | 48.3 ± 20.5 | 138.9 ± 27 | 115.7 ± 28 | 64 ± 23.8 |
|  |  | antennae | 11 | 0.1 ± 0.1 | 1.3 ± 0.5 | 1.6 ± 0.9 | 1.3 ± 0.9 | 0 | 4.5 ± 1.8 | 6.8 ± 3.7 | 5.3 ± 3.3 |
|  |  | ovipositor | 6 | 0.3 ± 0.3 | 1.8 ± 1 | 76.2 ± 74 | 0.5 ± 0.3 | 0.4 ± 0.4 | 78.6 ± 45.3 | 69.7 ± 39.1 | 5.8 ± 4.2 |
|  | linalool | stay | 20 | 10.3 ± 4.6 | 14.9 ± 9.4 | 6.4 ± 2.9 | 2.6 ± 0.6 | 128.3 ± 27.3 | 98.9 ± 28 | 90.3 ± 27 | 152 ± 30.4 |
|  |  | antennae | 15 | 2.2 ± 1 | 2.2 ± 1.5 | 1 ± 0.5 | 1.4 ± 0.4 | 2.3 ± 0.9 | 0.9 ± 0.7 | 4 ± 3.2 | 2.1 ± 0.8 |
|  |  | ovipositor | 8 | 1.8 ± 0.4 | 0.6 ± 0.4 | 0.5 ± 0.4 | 1.6 ± 0.5 | 53.9 ± 20.4 | 38.6 ± 33.8 | 32.9 ± 32.7 | 35.7 ± 17.2 |
|  | *(E)/(Z)*-linalool oxide (fur/pyr) | stay | 12 | 44.7 ± 19.1 | 17.4 ± 8.3 | 24.5 ± 14.1 | 13.3 ± 8.2 | 66 ± 23.4 | 46.6 ± 20.8 | 28.5 ± 12.7 | 84.1 ± 31.4 |
|  |  | antennae | 2 | 0 | 0 | 1.5 ± 0.5 | 0.5 ± 0.5 | 0 | 0 | 1.9 ± 1.1 | 0.9 ± 0.9 |
|  |  | ovipositor | 2 | 1 ± 1 | 1 ± 0 | 0 | 0 | 4.1 ± 4.1 | 66.7 ± 64.4 | 0 | 0 |
|  | methyl salicylate | stay | 17 | 8.2 ± 1.6 | 4.3 ± 0.8 | 7.3 ± 1.4 | 7.9 ± 1.9 | 88.7 ± 22.8 | 116.6 ± 31.5 | 44.1 ± 17.8 | 93.4 ± 20.8 |
|  |  | antennae | 16 | 1.1 ± 0.4 | 0.8 ± 0.4 | 1 ± 0.5 | 2.1 ± 0.7 | 1.9 ± 0.7 | 1 ± 0.5 | 1 ± 0.5 | 3.4 ± 1.3 |
|  |  | ovipositor | 10 | 1 ± 0.5 | 1.5 ± 1.2 | 0.4 ± 0.2 | 1.2 ± 0.5 | 17.9 ± 13.3 | 4.8 ± 4 | 1.2 ± 0.8 | 9.6 ± 4.7 |
|  | *(S)*-(-)-perillaldehyde | stay | 20 | 3.3 ± 0.6 | 2.5 ± 0.9 | 3.1 ± 0.8 | 2.5 ± 0.7 | 136.4 ± 31.9 | 120.1 ± 30.2 | 33.7 ± 18.4 | 74.9 ± 25.8 |
|  |  | antennae | 11 | 1.2 ± 0.6 | 1.1 ± 0.6 | 0.3 ± 0.2 | 0.9 ± 0.4 | 5 ± 2.7 | 5.2 ± 2.9 | 0.9 ± 0.7 | 2.6 ± 1 |
|  |  | ovipositor | 5 | 0.8 ± 0.6 | 1 ± 0.8 | 0.6 ± 0.6 | 2 ± 0.9 | 39.5 ± 39.2 | 19.7 ± 12.1 | 0.3 ± 0.3 | 73.4 ± 43.8 |
| *L. botrana* | *(E)*-β-caryophyllene | stay | 16 | 40.4 ± 6.9 | 42 ± 9.6 | 29.8 ± 5 | 34.1 ± 8.1 | 103 ± 11.7 | 134.1 ± 18.6 | 78 ± 15.7 | 98.6 ± 15.1 |
|  |  | antennae | 11 | 0.7 ± 0.2 | 0.9 ± 0.3 | 0.2 ± 0.2 | 0.6 ± 0.2 | 3.5 ± 1.2 | 3.4 ± 1.1 | 0.9 ± 0.9 | 2.7 ± 1.2 |
|  |  | ovipositor | 2 | 1 ± 0 | 1 ± 1 | 0 ± 0 | 2 ± 2 | 3.1 ± 2.9 | 20.7 ± 20.7 | 0 ± 0 | 2.4 ± 2.4 |
|  | (-)-α-cedrene | stay | 19 | 15.5 ± 2.6 | 21.2 ± 4.7 | 14 ± 2.4 | 16.3 ± 3.1 | 66 ± 12 | 75.6 ± 11.8 | 75.3 ± 11.6 | 67.4 ± 11.5 |
|  |  | antennae | 9 | 0.4 ± 0.2 | 0.3 ± 0.2 | 0.1 ± 0.1 | 0.8 ± 0.2 | 0.4 ± 0.2 | 0.6 ± 0.4 | 0.1 ± 0.1 | 0.7 ± 0.2 |
|  |  | ovipositor | 11 | 1.5 ± 0.8 | 2.6 ± 1.3 | 5.7 ± 1.7 | 2.4 ± 0.8 | 10 ± 6.6 | 16.3 ± 11.1 | 31.3 ± 8.9 | 8.5 ± 2.9 |
|  | cumene | stay | 14 | 22.1 ± 10.7 | 46.5 ± 22.1 | 35.6 ± 17.4 | 18.7 ± 9.8 | 49.9 ± 15.1 | 57.2 ± 17 | 129.2 ± 27.3 | 72.9 ± 20.9 |
|  |  | antennae | 5 | 0.6 ± 0.4 | 0.2 ± 0.2 | 1.2 ± 0.5 | 0.4 ± 0.4 | 0.7 ± 0.4 | 0.3 ± 0.3 | 2.4 ± 1.2 | 0.3 ± 0.3 |
|  |  | ovipositor | 4 | 0.5 ± 0.5 | 17.2 ± 14.1 | 3.8 ± 1.8 | 1 ± 1 | 0.5 ± 0.5 | 18.3 ± 16.8 | 37.6 ± 24.9 | 10.4 ± 10.4 |
|  | α/β-farnesene (mixture of isomers) | stay | 18 | 17.6 ± 5.1 | 2.2 ± 0.7 | 1.7 ± 0.7 | 6.8 ± 3.7 | 44.3 ± 19.4 | 40 ± 20.2 | 39.1 ± 20.9 | 44 ± 22.2 |
|  |  | antennae | 5 | 2.2 ± 1.2 | 1 ± 0.3 | 1.4 ± 1 | 3 ± 1.9 | 2.6 ± 1.2 | 1.9 ± 1.1 | 0.8 ± 0.5 | 1.2 ± 0.7 |
|  |  | ovipositor | 2 | 0 ± 0 | 1.5 ± 1.5 | 0.5 ± 0.5 | 0.5 ± 0.5 | 0 | 11.7 ± 11.7 | 0.2 ± 0.2 | 0.2 ± 0.2 |
|  | *(±)*-limonene | stay | 20 | 40.7 ± 9.8 | 40.8 ± 8.8 | 34.1 ± 8.7 | 29.1 ± 6.7 | 73.2 ± 14.8 | 62.6 ± 13.3 | 84.3 ± 13.8 | 93.1 ± 18.6 |
|  |  | antennae | 6 | 0.8 ± 0.3 | 0.3 ± 0.2 | 0.5 ± 0.2 | 1.2 ± 0.5 | 2.8 ± 1.2 | 0.7 ± 0.5 | 1.9 ± 1.1 | 3.4 ± 1.3 |
|  |  | ovipositor | 5 | 0.2 ± 0.2 | 0.2 ± 0.2 | 2 ± 1.1 | 2.8 ± 1.7 | 1.6 ± 1.6 | 0.4 ± 0.4 | 14.9 ± 9.2 | 27.7 ± 16.7 |
|  | linalool | stay | 16 | 4.8 ± 2.5 | 3.4 ± 0.8 | 2.4 ± 0.8 | 23.2 ± 18.4 | 26.4 ± 9.8 | 32.4 ± 10 | 7.7 ± 3 | 44.5 ± 17.9 |
|  |  | antennae | 6 | 0.2 ± 0.2 | 0.3 ± 0.2 | 0.2 ± 0.2 | 3.8 ± 2.3 | 0.3 ± 0.3 | 0.9 ± 0.8 | 0.5 ± 0.5 | 7.6 ± 3.1 |
|  |  | ovipositor | 6 | 0.5 ± 0.3 | 1 ± 0.5 | 0.2 ± 0.2 | 0.3 ± 0.3 | 5.9 ± 4.8 | 32.7 ± 21.3 | 0.1 ± 0.1 | 0.9 ± 0.9 |
|  | *(E)/(Z)*-linalool oxide (fur/pyr) | stay | 8 | 14.1 ± 7.8 | 14.4 ± 7.9 | 20.9 ± 12.8 | 18.6 ± 12.7 | 84 ± 42.8 | 20 ± 11.5 | 32 ± 20.6 | 78.7 ± 36.9 |
|  |  | antennae | 2 | 0 ± 0 | 1.5 ± 1.5 | 2 ± 0 | 1.5 ± 0.5 | 0 | 2.3 ± 2.3 | 3.4 ± 2.2 | 3.1 ± 2.4 |
|  |  | ovipositor | 3 | 3.3 ± 0.9 | 0 ± 0 | 0.3 ± 0.3 | 3.3 ± 3.3 | 42.1 ± 24.5 | 0 | 0.5 ± 0.5 | 38.3 ± 38.3 |
|  | methyl salicylate | stay | 19 | 32.3 ± 7.8 | 30.1 ± 6.2 | 32.1 ± 8.7 | 52.5 ± 17.6 | 59.9 ± 14 | 70.2 ± 19.1 | 56.2 ± 15.3 | 82.5 ± 14.9 |
|  |  | antennae | 8 | 0.5 ± 0.3 | 0.2 ± 0.2 | 2.1 ± 1.7 | 2.5 ± 1.1 | 2.2 ± 1.4 | 0.6 ± 0.5 | 1.5 ± 0.9 | 4.1 ± 1.5 |
|  |  | ovipositor | 8 | 1.6 ± 0.8 | 2.1 ± 1 | 2.2 ± 1 | 1.2 ± 0.6 | 10.4 ± 6.8 | 13.8 ± 9.1 | 17.1 ± 10.6 | 5.4 ± 3.8 |
|  | *(S)*-(-)-perillaldehyde | stay | 15 | 29.8 ± 5 | 42 ± 9.6 | 40.4 ± 6.9 | 34.1 ± 8.1 | 56.3 ± 19.4 | 73.2 ± 21.2 | 80.3 ± 22 | 72.3 ± 20.9 |
|  |  | antennae | 7 | 0.2 ± 0.2 | 0.9 ± 0.3 | 0.7 ± 0.2 | 0.6 ± 0.2 | 1.2 ± 0.5 | 0.8 ± 0.8 | 1 ± 0.7 | 2.9 ± 1.5 |
|  |  | ovipositor | 3 | 0 | 1 ± 1 | 1 ± 0 | 2 ± 2 | 0 | 0.6 ± 0.6 | 1.3 ± 1.3 | 1.1 ± 1.1 |

Table S2. Summary of statistical parameters explaining the duration spent by *E. ambiguella* or *L. botrana* within the olfactometer arena according to GLM and F-test (*p<0.05; ***p<0.001). Durations may be explained by the factors ‘volatile sector’^1^ or ‘behavior’^2^

| **VOC** | **factor** | ***E. ambiguella*** | | | | | ***L. botrana*** | | | | |
| --- | --- | --- | --- | --- | --- | --- | --- | --- | --- | --- | --- |
|  |  | **Df1** | **Df2** | **F-value** | **p-value** | | **Df1** | **Df2** | **F-value** | **p-value** | |
| *(E)*-ß-caryophyllene | sector | 3 | 154 | 0.63 | 0.6 | n.s. | 3 | 110 | 3,68 | 0.05 | n.s. |
|  | behavior | 2 | 154 | 26.08 | 1.76x10^-10^ | *** | 2 | 110 | 193,98 | 2.00x10^-16^ | *** |
| (-)-α-cedrene | sector | 3 | 116 | 0.47 | 0.7 | n.s. | 3 | 144 | 1,05 | 0.37 | n.s. |
|  | behavior | 2 | 116 | 30.37 | 2.47x10^-11^ | *** | 2 | 144 | 106,02 | 2.00x10^-16^ | *** |
| cumene | sector | 3 | 152 | 0.55 | 0.64 | n.s. | **3** | **80** | **4,03** | **0.01** | ***** |
|  | behavior | 2 | 152 | 37.54 | 5.62x10^-14^ | *** | 2 | 80 | 47,12 | 3.00x10^-14^ | *** |
| α/β-farnesene (mixture) | sector | **3** | **166** | **7.84** | **6.36x10^-5^** | ******* | 3 | 138 | 0,72 | 0.53 | n.s. |
|  | behavior | 2 | 166 | 21.53 | 4.88x10^-9^ | *** | 2 | 138 | 3,32 | 0.04 | * |
| (±)-limonene | sector | **3** | **130** | **6.68** | **3.01x10^-4^** | ******* | 3 | 118 | 2,84 | 0.05 | n.s. |
|  | behavior | 2 | 130 | 26.95 | 1.62x10^-10^ | *** | 2 | 118 | 99,66 | 2.00x10^-16^ | *** |
| linalool | sector | 3 | 166 | 2.93 | 0.05 | n.s. | 3 | 106 | 2,71 | 0.05 | n.s. |
|  | behavior | 2 | 166 | 38.28 | 2.13x10^-14^ | *** | 2 | 106 | 9,01 | 2.04x10^-4^ | *** |
| *(E)/(Z)*-linalool oxide (pyr/fur) | sector | 3 | 58 | 0.94 | 0.43 | n.s. | **3** | **46** | **11,47** | **0.02** | ***** |
|  | behavior | 2 | 58 | 7.68 | 1.10x10^-3^ | *** | 2 | 46 | 2,47 | 0.09 | n.s. |
| methyl salicylate | sector | 3 | 166 | 2.73 | 0.05 | n.s. | 3 | 134 | 0,54 | 0.66 | n.s. |
|  | behavior | 2 | 166 | 73.69 | 2.00x10^-16^ | *** | 2 | 134 | 65,16 | 2.10x10^-16^ | *** |
| (S)-(-)-perillaldehyde | sector | 3 | 116 | 3.50 | 0.06 | n.s. | **3** | **106** | **6,67** | **3.59x10^-4^** | ******* |
|  | behavior | 2 | 116 | 11.77 | 1.90x10^-5^ | *** | 2 | 106 | 5,62 | 4.78x10^-3^ | *** |

Bold values represent VOCs influencing *E. ambiguella* or *L. botrana*.

^1^4 levels: DCM, reference 1 (air), reference 2 (air) and VOC

^2^3 levels: stay, antennae-activity and ovipositor-activity
